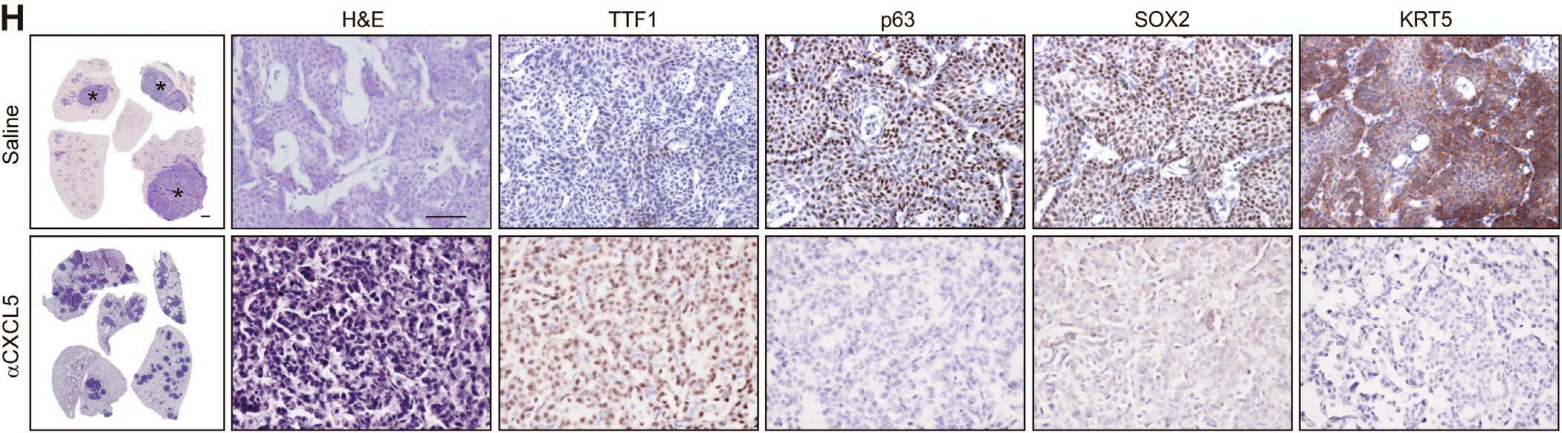# Correction: TET2–STAT3–CXCL5 nexus promotes neutrophil lipid transfer to fuel lung adeno-to-squamous transition

**DOI:** 10.1084/jem.2024011106032024c

**Published:** 2024-06-07

**Authors:** Yun Xue, Yuting Chen, Sijia Sun, Xinyuan Tong, Yujia Chen, Shijie Tang, Xue Wang, Simin Bi, Yuqin Qiu, Qiqi Zhao, Zhen Qin, Qin Xu, Yingjie Ai, Leilei Chen, Beizhen Zhang, Zhijie Liu, Minbiao Ji, Meidong Lang, Luonan Chen, Guoliang Xu, Liang Hu, Dan Ye, Hongbin Ji

Vol. 221, No. 7 | https://doi.org/10.1084/jem.20240111 | May 28, 2024

*JEM* regrets that an old version of the saline H&E image in Fig. 2 H was used in the originally published article due to a production error. The corrected panel is shown here. This correction does not change the original conclusions of the article, and the figure legend remains unchanged. The error appears in PDFs downloaded before June 3, 2024.

**Figure:**